# Supplementary material for: Identification of photoperiod-induced specific miRNAs in the adrenal glands of Sunite sheep (Ovis aries)
Source: Front Vet Sci. 2022 Jul 22;9:888207. doi: 10.3389/fvets.2022.888207 (PMC9354845; doi:10.3389/fvets.2022.888207)
Supplement: Supplementary file 1 [file Data_Sheet_1.ZIP › Supplementary file/miRDeep2 file.zip!/miRDeep2 file/Novel_Structure/SPLP42A1/Novel_460.pdf]

Provisional ID	: NC_019464.2_12687
Score total	: 2.2
Score for star read(s)	: -1.3
Score for read counts	: 0
Score for mfe	: 1.9
Score for randfold	: 1.6
Score for cons. seed	:
Total read count	: 26
Mature read count	: 26
Loop read count	: 0
Star read count	: 0

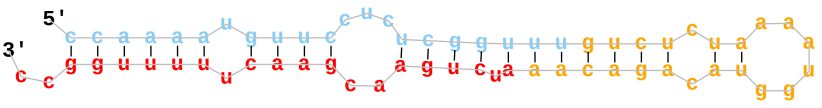

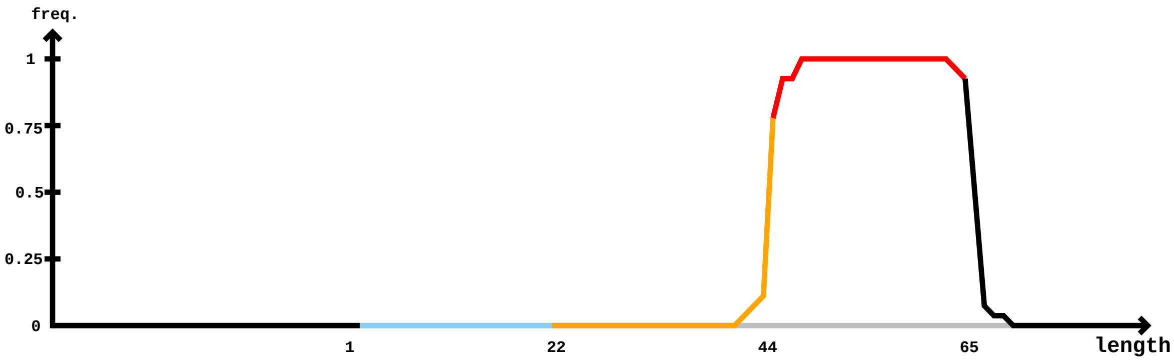

Star Mature

```
5'- aaacugaccuuuuccauuguauuggguuggccaaaauguuccucucgguuugucucuaaaaugguacagacaaaucugaacgaacuuuuuggccaacccaaacuuccuugga  -3'   exp
    ............((((..((.(((((((((((((((.((((...(((((((((((.((......)).))))))).))))..)))).)))))))))))))))))).....))))    reads   mm   sample
    ......................................................................caaaucugaacgaacuuuuugg....................    1       0    seq
    .......................................................................aaaucugaacAaacuuuuuggcc..................    1       1    seq
    ........................................................................aaucugaacAaacuuuuuggc...................    1       1    seq
    .........................................................................aucugaacAaacuuuuuggcc..................    18      1    seq
    ..........................................................................ucugaacAaacuuuuuggcca.................    4       1    seq
    ............................................................................ugaacgaacuuuuuggccaa................    1       0    seq
    ............................................................................ugaacgaacuuuuuggccaacU..............    1       1    seq
```
